# FEASIBILITY AND ACCEPTABILITY OF USING PLASMA BIOMARKERS FOR DIAGNOSING ALZHEIMER’S DISEASE IN PRIMARY CARE

**DOI:** 10.1093/geroni/igad104.1568

**Published:** 2023-12-21

**Authors:** Nicole Fowler, Kristen Swartzell, Dustin Hammers, Jared Brosch, James Murray, Deanna Willis

**Affiliations:** Indiana University School of Medicine, Indianapolis, Indiana, United States; Indiana University School of Medicine, IU Center for Aging Research, Indianapolis, Indiana, United States; Indiana University, Indianapolis, Indiana, United States; Indiana University, Indianapolis, Indiana, United States; Davos Alzheimer’s Collaborative, Wayne, Pennsylvania, United States; Indiana University School of Medicine, Indianapolis, Indiana, United States

## Abstract

Blood-based biomarkers (Aβ, P-tau, neurofilament light) are clinically available to aid in the diagnosis of Alzheimer’s disease and related dementias (ADRD). However, no research has examined the use of blood biomarkers to aid in the diagnosis of ADRD in primary care (PC). Our study will test feasibility and acceptability of implementing blood-based biomarkers for ADRD in PC. Participants include: all PC patients ≥65 years presenting to one of six PC clinics between 6/1/22 and 5/31/23 who score cognitively impaired on the Linus Health Digital Clock and Recall (DCR™), and PC providers (PCPs) of these patients. Patients will view a decision guide about biomarkers and complete the Concerns about Alzheimer’s Disease Dementia Scale, and the Future Time Perspective Scale and the Impact of Events Scale. These measures and the PHQ-9 and GAD-7 will be repeated within 2 weeks of results disclosure. PCPs will receive training on biomarker disclosure techniques and best practices. To date 9 PCPs have consented to provide the biomarker results disclosure and 11 have declined. Following completion of PCP consent (n=100), a total of 200 patients who failed the DCR are eligible to be approached for consent. By November 2023, we anticipate that 150 patients will have completed biomarker testing, and we will have examined the biomarker results in the context of patient neuropsychological and clinical data, comorbidities, and sociodemographic information. This study will provide information regarding feasibility and utility of ADRD biomarkers in PC and a preliminary analysis of biomarker results compared with the patients’ clinical profiles.

